# Low Discrepancy Sparse Phased Array Antennas

**DOI:** 10.3390/s21237816

**Published:** 2021-11-24

**Authors:** Travis Torres, Nicola Anselmi, Payam Nayeri, Paolo Rocca, Randy Haupt

**Affiliations:** 1 Electrical Engineering Department, Colorado School of Mines, Golden, CO 80401, USA; travistorres@mines.edu; 2 Consorzio Nazionale Interuniversitario per le Telecomunicazioni (CNIT), “University of Trento” ELEDIA Research Unit, via Sommarive 9, 38123 Trento, Italy; nicola.anselmi.1@unitn.it (N.A.); paolo.rocca@unitn.it (P.R.); 3 Haupt Associates, Boulder, CO 80303, USA; randyhaupt@gmail.com

**Keywords:** phased array, sensor array, sparse array, nonuniform array, planar array, random array, low discrepancy sequence

## Abstract

Sparse arrays have grating lobes in the far field pattern due to the large spacing of elements residing in a rectangular or triangular grid. Random element spacing removes the grating lobes but produces large variations in element density across the aperture. In fact, some areas are so dense that the elements overlap. This paper introduces a low discrepancy sequence (LDS) for generating the element locations in sparse planar arrays without grating lobes. This nonrandom alternative finds an element layout that reduces the grating lobes while keeping the elements far enough apart for practical construction. Our studies consider uniform sparse LDS arrays with 86% less elements than a fully populated array, and numerical results are presented that show these sampling techniques are capable of completely removing the grating lobes of sparse arrays. We present the mathematical formulation for implementing an LDS generated element lattice for sparse planar arrays, and present numerical results on their performance. Multiple array configurations are studied, and we show that these LDS techniques are not impacted by the type/shape of the planar array. Moreover, in comparison between the LDS techniques, we show that the Poisson disk sampling technique outperforms all other approaches and is the recommended LDS technique for sparse arrays.

## 1. Introduction

Sensing applications, such as radio telescopes, satellite communications, sonars, and defense radars, require large antenna arrays. Physically large arrays offer high resolution as well as high directivity (as long as the element spacing, or sampling remains small). Since the array cost is proportional to the number of elements in the array, designers try to minimize the number of elements in the aperture. However, if the array has a uniform grid of elements that undersamples the aperture (large element spacing), then grating lobes (extra main beams) result due to aliasing and are predictable from theory [1]. Most arrays are designed with an element spacing of $\lambda /\left(1+\sin\theta_{\max}\right)$ or less, where λ is the wavelength and $\theta_{\max}$ is the maximum scan angle from broadside.

Thinned and aperiodic arrays have fewer elements than dense periodic arrays [2,3,4]. For a dense array, thinning (removing elements from a regular grid [5]) and aperiodic spacing (spacing between elements are not constant [6]) mimic low sidelobe amplitude distributions through an amplitude density across the aperture. These arrays have far field patterns with low sidelobes near the main beam and increased sidelobe levels farther from the main beam.

Sparse arrays fill an antenna aperture with elements that are widely separated from each other in order to reduce the cost but maintain a narrow beamwidth. The definition of a sparse array in both the antenna and signal processing literature is vague. For instance, the IEEE standard defines a sparse antenna array as [7]: “An array antenna that contains substantially fewer driven radiating elements than a conventional uniformly spaced array with the same beamwidth having identical elements. Interelement spacings in the sparse array can be chosen such that no large grating lobes are formed and sidelobes are reduced.” On the other hand, mathematics has a different definition: a sparse matrix has most elements equal to zero [8]. Sparsity of a matrix equals the number of zero valued elements divided by the total number of elements. The words “fewer” and “most” do not specify a sharp dividing line between dense and sparsity. It is important to note here that while these two definitions of sparsity are different, both have been used in designing sparse arrays. For the latter, compressive sensing (CS) approaches aim at solving a system of linear equations, forcing the solution to be maximally sparse, namely, to have the minimum number of nonzero coefficients, with respect to an expansion basis [9]. Accordingly, CS-based methods have been applied to the synthesis of sparse arrays by properly reformulating the design problem as a pattern matching one [10]. The problem unknowns are the set of complex (amplitude and phase) excitation coefficients of the “candidate” array elements, the positions of these latter obtained through a dense sampling of the array aperture. The CS solution is the sparse complex-valued vector of the excitations, and the positions of the array elements are obtained as a byproduct and correspond to the candidate locations having non-null coefficient [10]. CS has been applied to the design of both linear [11,12] and planar [13,14] sparse arrays, considering symmetric [11,13] as well as asymmetric [12,14] pattern shapes. The obtained results have shown achieving up to 40% elements reduction with respect to regular/uniform array arrangements. It is important to note that CS approaches do not change the definition of sparse arrays; but deal with minimizing the number of nonzero coefficients in the system of equations in designing the array.

The authors of this paper propose the following definition of sparsity, based on the IEEE definition [7], but we add that: a sparse array has an average element spacing greater than λ. If the array has a uniform square grid of isotropic elements, then grating lobes exist when the main beam points at broadside. Periodic sparse arrays have grating lobes with the same gain as the main beam. A random distribution of elements in the sparse array lowers the grating lobes to a level of the surrounding sidelobes. Low sidelobes are not an option for sparse arrays.

**Figure 1:**
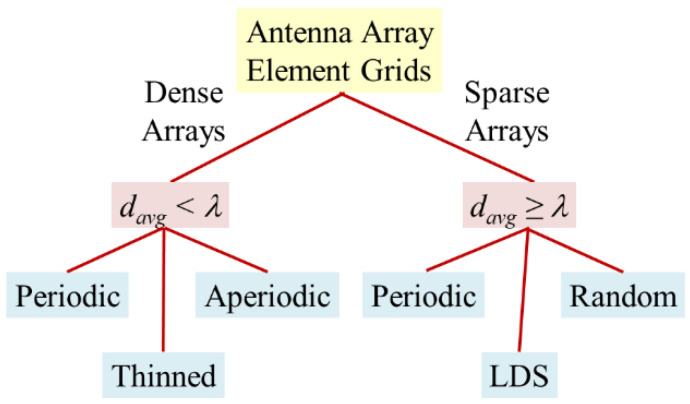
Categories of antenna array grids.

Figure 1 distinguishes between dense and sparse arrays. Given this definition, our literature review of sparse arrays emphasizes papers that present arrays with an average element spacing of at least one wavelength. Sparse arrays (by our definition) in the literature generally have random element spacing [15,16,17].

Sparse arrays are widely used in radio telescopes and MIMO systems, as well as other sensing systems. The Long Wavelength Array Station 1 (LWA1) is an aperture synthesis imaging array (20–80 MHz) for radio astronomy [18]. It contains 512 antenna elements inside an ellipse that has a 110 m major axis and a 100 m minor axis. To reduce grating lobes in this sparse array at 80 MHz, the elements are pseudorandomly distributed with a 5 m minimum spacing constraint that results in an average element spacing of about 5.4 m or 1.44*λ* at 80 MHz. The LOw Frequency ARray (LOFAR) Low Band Antenna (LBA) in the Square Kilometer Array (SKA) consists of 96 dual polarized crossed dipole active antennas operating from 30 to 80 MHz [19]. An inner array of 46 randomly spaced dual-polarized elements have average element spacings between 0.4*λ* and 0.8*λ* within a 30 m radius, while the outer array of 48 randomly spaced dual-polarized elements have average element spacings between 0.8*λ* and 1.7*λ* within an annulus having an inner diameter of 30 m and outer diameter of 85 m [20].

Some examples of very sparse radio telescopes include the Very Long Baseline Array (VLBA) that has 10 parabolic reflectors that are 25 m in diameter forming a total collecting area of 19,635 m^2^ [21], The Atacama Large Millimeter/submillimeter Array (ALMA) that has 10 parabolic reflectors that are 12 m in diameter forming a total collecting area of 6600 m^2^ [22], and the Very Large Array (VLA) that has 27 parabolic reflectors that are 25 m that extend in a “Y” shape with each arm over 15 km long [23].

Microwave imaging experiments were performed using an array aperture of 320 × 320 mm operating over 17–20 GHz [24]. The first model was a fully populated planar array of 64 × 64 elements on a square grid. The second model was a sparse array of randomly placed elements. An x-y positioner moved a single antenna to the designated element positions in the aperture to form the dense and sparse arrays. A compressive sensing algorithm (average sampling rate that is less than the Nyquist rate) outperformed other reconstruction algorithms for sparse arrays having 1024, 400, and 160 antennas which correspond to 25%, 10%, and 4% of the elements in the fully populated array.

Sparse antenna arrays with random element spacing significantly improve the sum rate capacity (maximum aggregation of all the users’ data rates) of a MIMO base station antenna system [25]. The sparse array aperiodicity spreads the grating lobe (GL) energy over all the lower sidelobes [26].

The difference between the uniform element grid and the randomized element grid is mathematically known as the discrepancy. Using a low discrepancy sequence (LDS) to place elements in an array aperture ensures that elements do not overlap while keeping a uniform sampling of the aperture. An LDS produces a random-like equidistribution of elements using a deterministic generating formula. Equidistributed means that if the aperture is divided into equal subareas, then the number of elements in all subareas is the same. The aperture discrepancy approaches zero as the number of elements approaches infinity. Random element spacing has the highest discrepancy, because large areas of empty space as well as high densities of elements exist within the aperture. In contrast, elements on a regular grid have the lowest possible discrepancy. A low discrepancy element distribution appears random, but the elements also appear to be evenly distributed across the aperture.

Discrepancy theory has its origin in a paper by H. Weyl on the uniform distribution of sequences [27]. Different LDSs have been introduced from the early 1960s, including the Hammersley point set [28], the Sobol sequence [29], the Faure sequence [30], and the Niederreiter sequence [31]. LDSs found their first applications in the 1990s for numerical analysis and integration for numerical simulation, in the fields of computer graphics [25], computational physics [32], and finance engineering [33]. The application of LDS to the generation of sample points for Monte Carlo sampling (i.e., the quasi-Monte Carlo approach) is theoretically superior to a standard Monte Carlo technique.

To our knowledge, the first paper to apply an LDS (Hammersley sequence) to element spacing explored sparse aperiodic spacing on a spherical array [34]. The advantages of LDS for sparse phased array design demonstrated that the Hammersley sequence maintains the large separation between the elements, while reducing the grating lobes compared to element spacings derived from random, pseudo-random, and uniform plus jitter sequences [35].

In this paper we use low discrepancy sequences to distribute elements in a sparse planar array aperture while maintaining the following properties:

- Sufficient elements to achieve a desired gain.
- Aperture size is large enough to achieve the desired beamwidth.
- Elements have a minimum separation distance, so they can physically fit into the aperture and mutual coupling is not a problem.
- No GLs present at maximum scan angles.
- The average element spacing is greater than λ.

We study sparse LDS arrays with 86% less elements than a fully populated array, and present numerical results that demonstrate these sampling techniques are capable of completely removing the grating lobes of sparse arrays. We recall that that a fully populated array has a uniform spacing of half-wavelength between its elements. The mathematical formulation for implementing LDS generated element lattice for sparse planar arrays is presented. Multiple array configurations are also studied, and we show that these LDS techniques are not impacted by the type/shape of the array aperture. Finally, we show that in comparison between the LDS techniques, Poisson disk sampling technique outperforms all other approaches and is the recommended LDS technique for sparse arrays.

This paper is organized as follows. In Section 2, we describe the mathematical formulation of the LDS sampling techniques. In Section 3 we implement these techniques on a large square planar array with an aperture size of 32*λ* × 32*λ*. We then extend this study to other array types in Section 4. Beam scanning performance of the arrays is studied in Section 5, followed by conclusions in Section 6.

## 2. Sampling Points on a Planar Aperture

Assume a uniformly weighted *N*-element planar array lies in the x-y plane bounded by $0\leq x\leq x_{\max}$ and $0\leq y\leq y_{\max}$. The array factor is given by

(1) $$AF(u,v)=\sum_{n=1}^{N}e^{-jk\left[x_{n}u+y_{n}v\right]}$$

where k=2π/λ, λ is the wavelength, $\left(x_{n},y_{n}\right)$ is the location of element *n*, θ and ϕ are the elevation and azimuth angle, respectively, and u=sinθcosϕ, v=sinθsinϕ. We note that for a planar array, regular sampling takes the form of a rectangular lattice, triangular lattice, or concentric ring array. In this paper the rectangular lattice serves as the reference, where the grating lobes for a rectangular lattice appear at [1]

(2) $$\begin{array}{l} u_{m}=u_{s}+m\lambda /d_{x}\;\mathrm{for}\;m=0,\pm 1,\pm 2,\ldots ,\\ v_{n}=v_{s}+n\lambda /d_{y}\;\mathrm{for}\;n=0,\pm 1,\pm 2,\ldots , \end{array}$$

where *u_s_* and *v_s_* are the main beam location in the sine space.

### 2.1. Random Sampling Approaches

#### 2.1.1. Random Sampling

The element locations on a random lattice are defined by

(3) $$\left(x_{n},y_{n}\right)=\left(\alpha_{n}x_{\max},\beta_{n}y_{\max}\right)$$

where $\alpha_{n}$ and $\beta_{n}$ are uniformly distributed random variables between 0 and 1, and $x_{\max}$ and $y_{\max}$ are the maximum lattice size in *x* and *y*. Random element sampling is hierarchical, because adding an additional element does not require recalculating the previous element locations.

#### 2.1.2. Random Sampling with Jitter

Jitter adds a small random variation to the element location given by

(4) $$\left(x_{n},y_{n}\right)=\left({x^{\prime }}_{n}^{}+\alpha_{n}r_{\max}\cos\left(2\pi \beta_{n}\right),{y^{\prime }}_{n}^{}+\alpha_{n}r_{\max}\sin\left(2\pi \beta_{n}\right)\right)$$

where $\left({x^{\prime }}_{n}^{},{y^{\prime }}_{n}^{}\right)$ are the rectangular lattice coordinates and $r_{\max}$ is the maximum distance that the new sample point moves from the regular lattice.

#### 2.1.3. Random Hyperuniform Spatial Arrangements

Another important category of random distributions is random hyperuniform spatial arrangements which have been observed in different physical systems ranging from disordered ground state to jammed particle packing [36,37,38]. Hyperuniform systems are exotic states of matter which exploit designed disorder laying between a crystal and a liquid. A statistically homogeneous hyperuniform points configuration in *d*-dimensions is one in which the number variance of *N* points within a spherical observation window of radius *R* grows more slowly than *R^d^*, i.e.,

(5) $$\sigma_{N}^{2}(R)∼R^{d-1}$$

This is equivalent to having a structure factor that tends to zero as the wavenumber tends to zero, implying that single scattering of incident radiation at infinite wavelengths is completely suppressed, i.e., they do not have Bragg peaks. From an array sampling point perspective, these types of distributions can eliminate grating lobes. Due to the random nature of these distributions, we would limit our discussions on these hyperuniform arrangements and focus on LDS techniques and refer the interested reader to [36,37,38,39,40].

### 2.2. Low Discrepancy Sampling Approaches

**Figure 2:**
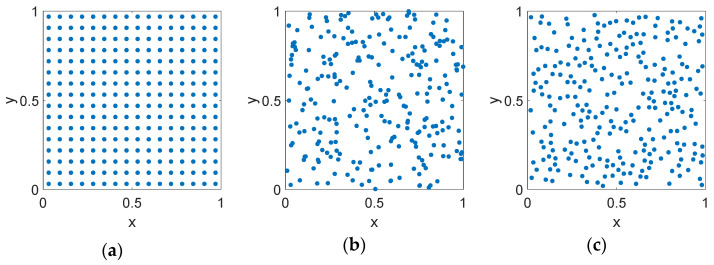
A generic representation of the position of points on a unit-square with different distributions: (**a**) uniform, (**b**) random, (**c**) LDS Poisson disk. The total number of elements is equal in all three cases but note that the LDS method fills the space more uniformly while avoiding too close spacings.

A low discrepancy sequence (LDS) is a set of points positioned on a surface that fills the aperture more uniformly than an equal set of uncorrelated random points [41]. Figure 2 shows the points on the x-y plane of a unit square for the case of uniform, random, and LDS Poisson disk distributions.

#### 2.2.1. Hammersley Sampling

Any positive integer can be represented by a prime base as

(6) $$n=\sum_{k=0}^{L(n)}a_{k(n)}b^{k}$$

where, *b* is the prime base in which number *n* is represented, $a_{k}(n)$ is an integer in [0,b−1], and L(n) is the lowest integer value that allows expressing the integer *n* in base *b* as the summation of L(n)+1 terms. The van der Corput sequence [42,43], is a one-dimensional low-discrepancy sequence over the unit-interval and is given by

(7) $$\Psi_{b}(n)=\sum_{k=0}^{L(n)}a_{k(n)}b^{-k-1}$$

For a binary representation, i.e., base 2, the van der Corput sequence can be written as

(8) $$\Psi_{2}(n)=\frac{a_{0}}{2}+\frac{a_{1}}{2^{2}}+\ldots +\frac{a_{L(n)}}{2^{L(n)+1}}$$

Hammersley sampling utilizes the van der Corput sequence that results in a two-dimensional LDS sampling in the rectangular grid defined by

(9) $$\left(\alpha_{n},\beta_{n}\right)=\left(\frac{n}{N},\Psi_{b}\left(n\right)\right),n=0,1,2,\ldots ,N-1.$$

Hamersley sampling is not hierarchical due to the n/N term in (8), because adding an additional element, does require recalculating the previous element locations.

#### 2.2.2. Halton Sampling

Halton sampling replaces the n/N term in Hamersley sampling by another Van der Corput sequence,

(10) $$\left(\alpha_{n},\beta_{n}\right)=\left(\Psi_{b{\;}_{1}}\left(n\right),\Psi_{b{\;}_{2}}\left(n\right)\right),n=0,1,2,\ldots ,N-1,$$

where *b*_1_ and *b*_2_ are two different prime bases. Note that Halton sampling is hierarchical.

#### 2.2.3. Sobol Sampling

The *j*th coordinate of the *i*th point, p(*i*, *j*), in a Sobol sequence [44,45,46,47], is given by

(11) $$\left\{ \begin{array}{ll} 0, & i=1\\ \gamma_{i}(1)v_{j}(1)⊕\gamma_{i}(2)v_{j}(2)⊕\ldots , & i>1. \end{array}\right.$$

Here ⊕ is the bitwise exclusive or operator, $\gamma_{i}(n)$ are the binary digits of the integer *i* − 1, and $v_{j}(n)$ are direction numbers that are generated from primitive polynomials in ${\mathbb{Z}}_{2}$. The direction numbers depend on the coordinate *j* and are obtained from primitive polynomials according to the *i*th dimension. The detailed process to determine these direction numbers can be found in [48]. Sobol sampling is hierarchical, since it does not depend on the total number of elements and adding additional elements does not require recalculation of element locations. For two-dimensional sampling with Sobol sequence, one option is to use the *i* points for one axis, and the *j* points for the orthogonal axis. This is similar to Hammersley sampling. Alternatively, one can use two Sobol sequences for the axes, similar to Halton sampling, which will improve the equidistribution.

#### 2.2.4. Poisson Disk Sampling

Poisson disk sampling produces points, *X* = {*x_i_*}, from a given domain, *D*, in *N*-dimensional space, that are tightly packed, but no closer than a specified minimum distance *r*. Here *N* is the number of elements in the array. The samples are at least a minimum distance apart, satisfying an empty disk criterion, i.e.,

(12) $$\forall x_{i},x_{j}\in X,x_{i}\neq x_{j}:\left\|x_{i}-x_{j}\right\|\geq r.$$

The maximal condition requires that the disks are simultaneously closely packed together, in the sense that the sample disks cover the whole domain. Mathematically this is given by

(13) $$\forall x\in D,\exists x_{i}\in X:\left\|x-x_{i}\right\|<r.$$

The Poisson distribution also possesses bias-free property which means that the expected number of sample points inside any subdomain, Ω, is proportional to the area of the subdomain. This is achieved by ensuring that the probability of selecting a point for the next sample is equal to the probability of selecting any other point, provided these points are not already inside some prior sample’s disk, i.e.,

(14) $$\forall x_{i}\in X,\;\forall \Omega \subset D_{i}:P\left(x_{i}\in \Omega \right)=\frac{\mathrm{Area}(\Omega )}{\mathrm{Area}(D_{i})}.$$

Multiple algorithms have been developed to implement this technique, and the reader is referred to [49,50] for detailed surveys of implementing Poisson sampling methods. Finally, we note that since this approach requires one to specify the total number of elements, it is not hierarchical.

## 3. Sparse Planar Phased Array Antennas

In this section, our example is a planar 32*λ* × 32*λ* aperture with 576 elements. Note that a fully populated array with *λ*/2 element spacing with this aperture size would require 4096 elements, so this sparse array removes 86% of the elements. With a uniform grid, the average element spacing of this sparse array is 4*λ*/3.

### 3.1. Element Distributions on the Aperture

**Figure 3:**
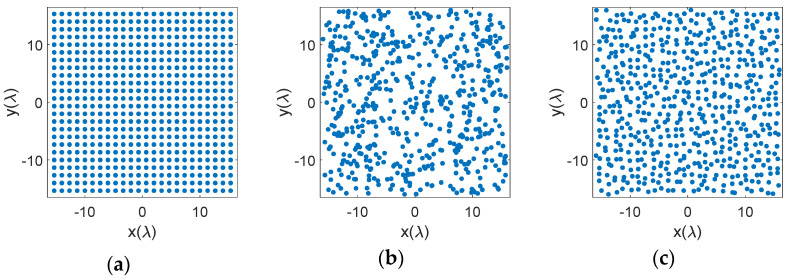

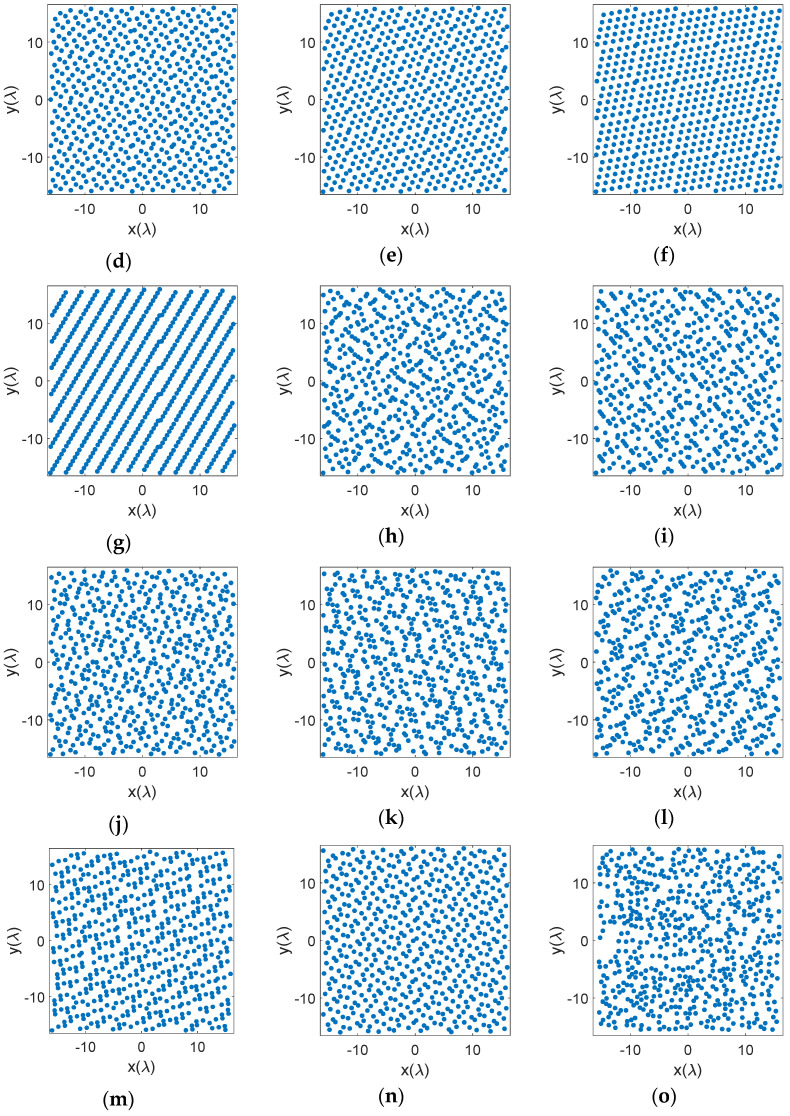
Position of elements on the aperture with different distributions: (**a**) uniform, (**b**) random, (**c**) random with jitter, (**d**) Hammersley (base 2), (**e**) Hammersley (base 3), (**f**) Hammersley (base 5), (**g**) Hammersley (base 7), (**h**) Halton (bases 2, 3), (**i**) Halton (bases 2, 5), (**j**) Halton (bases 2, 7), (**k**) Halton (bases 3, 5), (**l**) Halton (bases 3, 7), (**m**) Halton (bases 5, 7), (**n**) Sobol, (**o**) Poisson disk.

Examples of element distributions for uniform, random, and multiple LDS methods appear in Figure 3.

The LDS element placements in Figure 3d–o do not correspond to a periodic grid. Neither are they random. In comparison of the techniques, higher order Hammersley methods, Figure 3f,g with prime bases of 5 and 7 have shades of a periodic placement that potentially raise the peak sidelobe levels. This will be discussed further in the next section. Other LDS methods however appear more random and are suitable candidates for sparse arrays. Here we provide some metrics on the element distributions.

**Figure 4:**
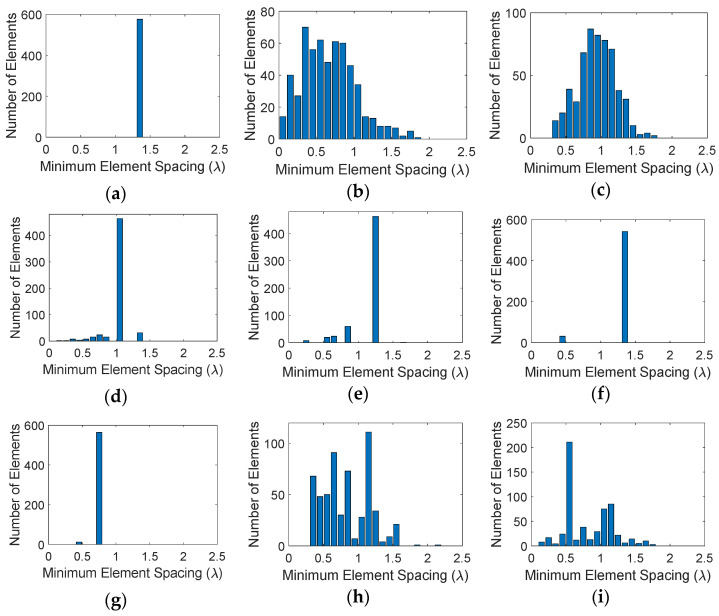

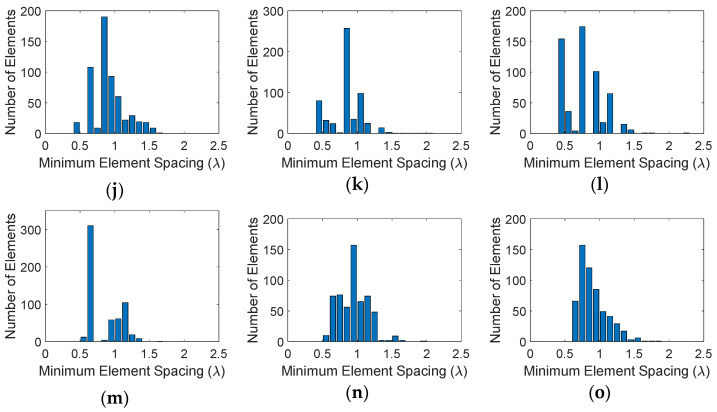
Bar plot of minimum element spacings with different distributions: (**a**) uniform, (**b**) random, (**c**) random with jitter, (**d**) Hammersley (base 2), (**e**) Hammersley (base 3), (**f**) Hammersley (base 5), (**g**) Hammersley (base 7), (**h**) Halton (bases 2, 3), (**i**) Halton (bases 2, 5), (**j**) Halton (bases 2, 7), (**k**) Halton (bases 3, 5), (**l**) Halton (bases 3, 7), (**m**) Halton (bases 5, 7), (**n**) Sobol, (**o**) Poisson disk.

As discussed earlier, from a practical fabrication perspective, we need elements distributed on the aperture in a manner that the physical antenna elements do not touch. Bar plots in Figure 4 show the number of elements that fall within a minimum range of element spacing for the different distributions. As expected, for the uniform case, Figure 4a, all elements have the same minimum separation between them. The random distribution, Figure 4b, on the other hand almost produces a Gaussian-like distribution of elements but has several elements that are placed too close to each other. Random distribution with jitter, Figure 4c, has a similar Gaussian-like distribution, but provides a slightly larger minimum element spacing. The distribution however looks too regular. Hammersley distributions, Figure 4d–g, place many of the elements at a certain minimum spacing, which avoids the issue with minimum element spacing, but the distributions appear more periodic. This is primarily due to the hierarchical problem with this sampling method. Halton samplings, Figure 4h–m, avoid both issues, i.e., too close placement of elements and a periodic distribution, although it appears that the performance improves as the base prime numbers are picked further away from each other, e.g., 2 and 7. Sobol and Poission samplings also show a Gaussian distribution of elements and avoid small element spacings. Notably, the Poisson distribution here was set to half the element spacing of the uniform array, i.e., 4λ/6, and it can be seen that the minimum element spacing is exactly 4λ/6 as designed. Note that Halton, Sobol, and Poisson distributions provide a non-uniform and well distributed placement of the elements, while avoiding small element distances.

### 3.2. Radiation Patterns of the Sparse Phased Array Antennas

The complete far-field radiation pattern of an antenna array with identical elements is given by

(15) $$F(u,v)=E(u,v)\sum_{n=1}^{N}a_{n}e^{-jk\left[x_{n}u+y_{n}v\right]}$$

where *E*(*u*, *v*) is the element pattern in u-v space, the summation represents the array factor, and *F*(*u*, *v*) is the far-field radiation pattern of the antenna array. In this expression, *a_n_* is the element weight, and the other terms are as defined in (1). Here we consider elements that have unity amplitude and zero phase, so *a_n_* = 1. The elements are also isotropic, so *E*(*u*, *v*) = 1.

**Figure 5:**
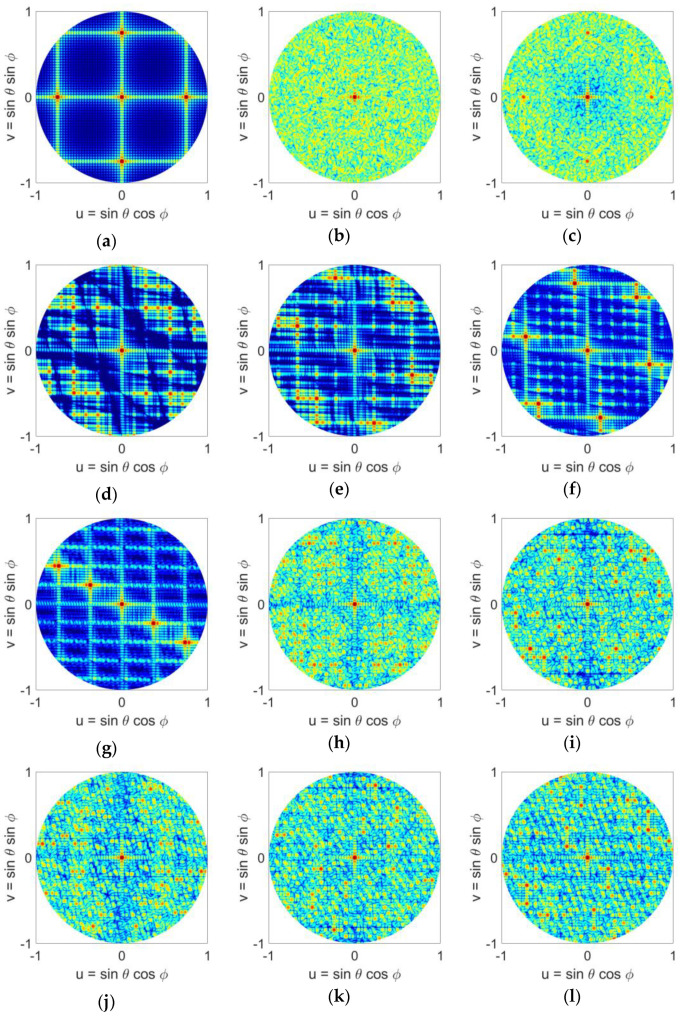

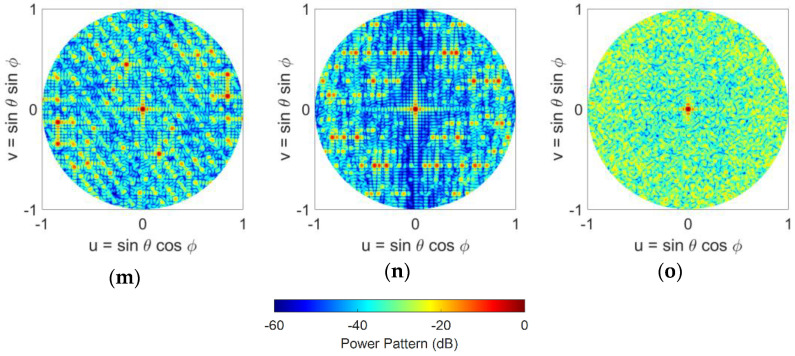
Normalized power patterns of the antenna arrays with different element distributions in the u-v space: (**a**) uniform, (**b**) random, (**c**) random with jitter, (**d**) Hammersley (base 2), (**e**) Hammersley (base 3), (**f**) Hammersley (base 5), (**g**) Hammersley (base 7), (**h**) Halton (bases 2, 3), (**i**) Halton (bases 2, 5), (**j**) Halton (bases 2, 7), (**k**) Halton (bases 3, 5), (**l**) Halton (bases 3, 7), (**m**) Halton (bases 5, 7), (**n**) Sobol, (**o**) Poisson disk.

The radiation patterns of the arrays in the u-v space are computed using (15) and are given in Figure 5. We note that in these graphs the visible region is a circle with $\sqrt{u^{2}+v^{2}}\leq 1$. It can be seen that the uniform case has the worst performance and four grating lobes with sidelobe level of 0 dB appear in the visible space. Random sampling completely removes the grating lobes, however as we saw in Section 2, it places many elements too close to each other. Random sampling with jitter reduces the grating lobes but cannot break the grating lobes completely because its element spacing is too regular. On the other hand, all LDS methods are effective in reducing the SLLs while maintaining low discrepancy. In comparison between these methods, Hammersley sampling has the poorest performance due to its hierarchical problem. For Hammersley sampling, performance degrades as the prime base number is increased. Halton sampling on the other hand shows a much better performance, and all 6 cases studied here show that they can break the grating lobes. Due to its binary implementation, Sobol sampling shows a similar performance to Hammersley sampling with a prime base of 2. Poisson sampling appears to outperform all other LDS methods as well as the random distribution. The 2D u-v graphs in Figure 5, allow one to visually compare the radiation performance of all the sampling techniques, however, for better comparison, a quantitative analysis is also provided in the next section. 

### 3.3. Quantitative Analysis of Sparse Array Performances

As discussed earlier in this work, for sparse arrays we are interested in removing the grating lobes, while at the same time having sampling that avoids too sparse or too dense element distributions. In order to quantitatively analyze these arrays, here we look at some metrics for element distributions and radiation pattern performances.

For element distributions, we look at an important statistical parameter, i.e., average minimum element spacing. This average value is obtained by computing the minimum element spacing for each element of the array, and then averaging the sum of those numbers over the total number of elements. These results are given in Table 1 for all 15 cases studied here. All LDS techniques provide a larger value of average minimum element spacing compared to the random technique, with Hammersley technique yielding the largest in comparison. From an element distribution perspective, it can be seen that LDS methods are very effective in providing a physically realizable distribution and outperform the random approach. For a quantitative study of radiation performance, we look at peak sidelobe level (SLL), as well as directivity and aperture efficiency. Peak SLL is the ratio of the pattern of the sidelobe peak (*F*_SLL_), to the pattern value of the main lobe (*F*_max_). We note that for a boresight beam *F*_max_ is the value of the far-field radiation pattern at *F*(0, 0). The directivity is defined as

(16) $$D=\frac{4\pi F_{\max}}{\int_{0}^{\sqrt{u^{2}+v^{2}}\leq 1}\int_{0}^{\sqrt{u^{2}+v^{2}}\leq 1}F(u,v)\;du\;dv}$$

Aperture efficiency is defined as directivity divided by maximum aperture directivity, where the maximum aperture directivity is given by

(17) $$D_{aperture}=\frac{4\pi A}{\lambda^{2}}$$

Here, *A* is the size of the array aperture, which in our study in this section is 32*λ* × 32*λ*, and *λ* is the wavelength.

These results are also given in Table 1 for all 15 cases. It can be seen that the uniform case has the poorest performance in terms of SLL as well as directivity and efficiency. Random distribution can notably improve these, but as discussed earlier, the element distribution is undesirable. Random distribution with jitter improves directivity and efficiency but degrades SLL. In comparison between the LDS methods, Hammersley sampling has the poorest SLL performance, which degrades as the prime base number is increased. Halton sampling shows a better performance in comparison. The prime bases of 2 and 7 yield the best performance for this technique. Sobol sampling shows a similar performance to Hammersley with a prime base of 2. Poisson disk sampling shows the best performance of all LDS techniques, with a SLL of −12.28 dB and close to 15% aperture efficiency. While all these LDS techniques outperform the uniform case, the best performances come from Halton and Poisson disk methods that outperform the random technique.

## 4. Aperture Shape Effects on the Performance of Sparse Phased Array Antennas

**Figure 6:**
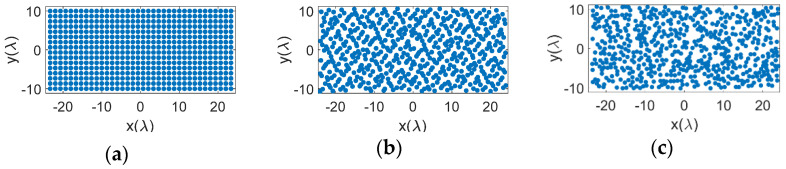

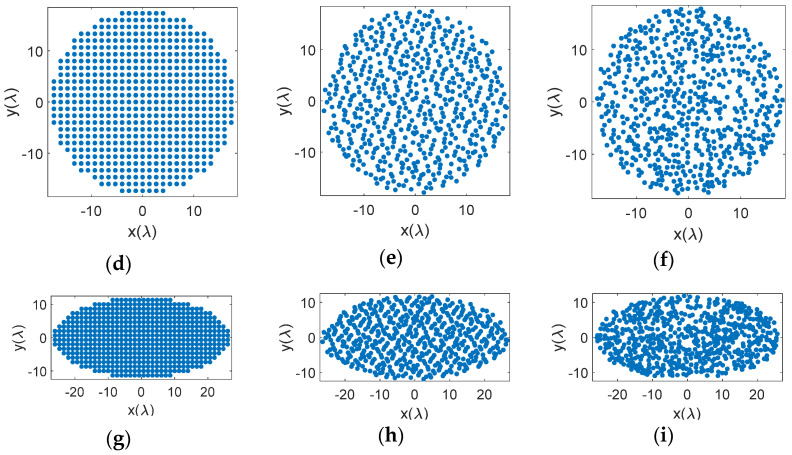
Position of elements on rectangular, circular, and elliptical apertures with different distributions: (**a**) uniform (rectangular), (**b**) Halton (rectangular), (**c**) Poisson disk (rectangular), (**d**) uniform (circular), (**e**) Halton (circular), (**f**) Poisson disk (circular), (**g**) uniform (elliptical), (**h**) Halton (elliptical), (**i**) Poisson disk (elliptical).

To see the impact of aperture shape on the performance of these LDS arrays, in this section we study three different types of arrays with rectangular, circular, and elliptical apertures. All apertures are designed with the same surface area of 1024*λ*^2^ as the square aperture studied in Section 3. The rectangular aperture has an aspect ratio of 9/4, i.e., the ratio of its longer side to its shorter side, corresponding to longer and shorter side lengths of 48*λ* and 64*λ*/3, respectively. The circular aperture has a radius of 18*λ*. To maintain the same aspect ratio as the rectangle and the same aperture size as the other arrays, the elliptical array major and minor axis are 27*λ* and 12*λ*, respectively. Here we compare the performance of two LDS sparse arrays that showed the best performance, namely Halton (with bases of 2 and 7) and Poisson distributions, with uniform distribution. The element distributions for these arrays are given in Figure 6.

**Figure 7:**
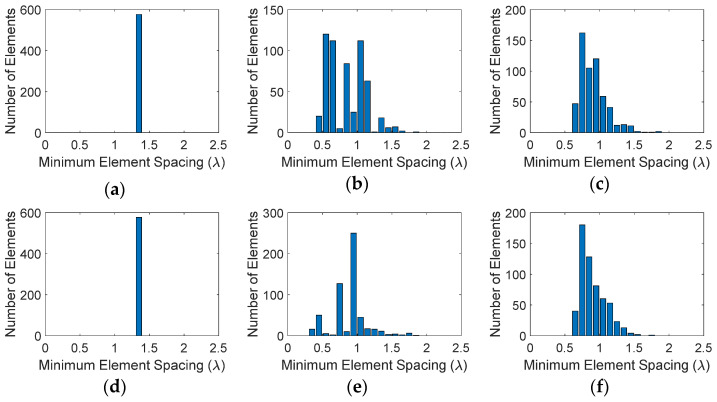

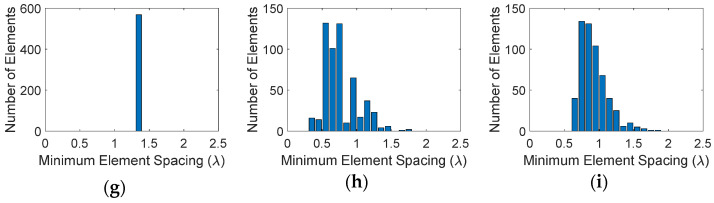
Bar plot of minimum element spacings on rectangular, circular, and elliptical apertures with different distributions: (**a**) uniform (rectangular), (**b**) Halton (rectangular), (**c**) Poisson disk (rectangular), (**d**) uniform (circular), (**e**) Halton (circular), (**f**) Poisson disk (circular), (**g**) uniform (elliptical), (**h**) Halton (elliptical), (**i**) Poisson disk (elliptical).

**Figure 8:**
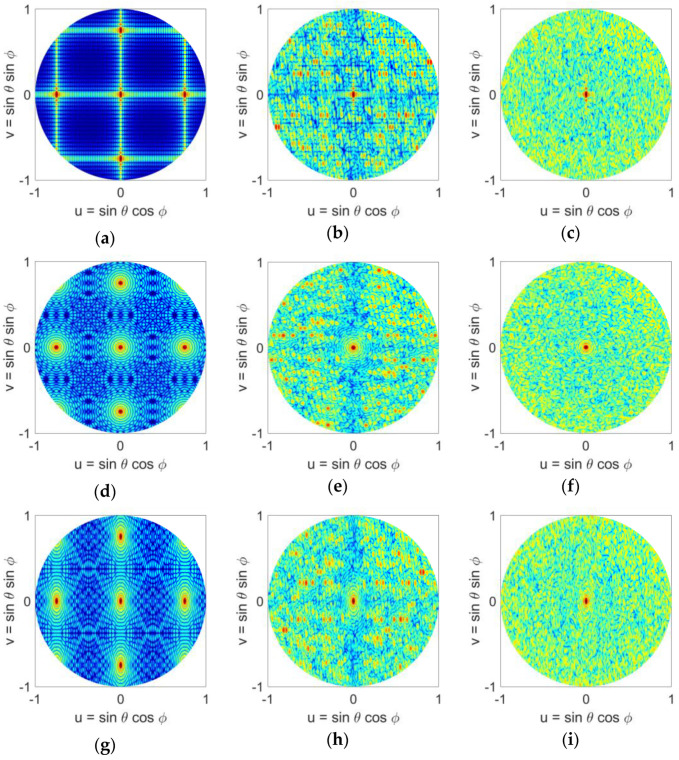
Normalized power patterns of sparse antenna arrays with rectangular, circular, and elliptical apertures and different element distributions in the u-v space: (**a**) uniform (rectangular), (**b**) Halton (rectangular), (**c**) Poisson disk (rectangular), (**d**) uniform (circular), (**e**) Halton (circular), (**f**) Poisson disk (circular), (**g**) uniform (elliptical), (**h**) Halton (elliptical), (**i**) Poisson disk (elliptical).

Bar plots in Figure 7 show the number of elements that fall within a minimum range of element spacing for the different distributions. Similar to Figure 4, for the uniform case, all elements have the same minimum separation between them. Halton sampling avoids too close placement of elements and distributes them in the range around λ/2 to 2λ, while Poisson sampling provides a Gaussian distribution of elements with and minimum element spacing of 4λ/6. We note that these observations are similar to the square aperture studies given in Section 3. The radiation patterns of these arrays in the u-v space are given in Figure 8, where it can be seen that similar to the square aperture array, the uniform case has the worst performance and four grating lobes with sidelobe level of 0 dB appear in the visible space. Both LDS techniques remove the grating lobes; however, it can be seen that the Poisson technique outperforms Hammersley. Nonetheless, these studies show that the performance of LDS sampling techniques are not impacted by the shape of the array aperture, and in general these sampling approaches can be used for arbitrary shaped arrays.

Table 2 summarizes the performance factors for these three types of arrays. As expected, the uniform case has the poorest performance in terms of SLL as well as directivity and efficiency. Both LDS techniques are effective in removing the grating lobes, but it can be seen that the Poisson technique provides the best results. It should be noted that in comparison between array aperture types, the circular aperture provides the best performance in terms of peak SLL, directivity, and efficiency.

## 5. Beam-Scanning Performance of Sparse Phased Array Antennas

In this section, we study the beam-scanning performance of sparse phased array antennas. The studies conducted in Section 3 and Section 4 showed that the performance of LDS distributions are not impacted by the type/shape of the array aperture, so for brevity we only consider the square aperture configuration in Section 3. By adding a progressive phase shift to the elements on the aperture, the sparse arrays can provide 2D beam scanning. Mathematically, the phase shift needed to scan the beam is given by

(18) $$\varphi (x_{n},y_{n})=k\left(x_{n}\sin\theta_{s}\cos\phi_{s}+y_{n}\sin\theta_{s}\sin\phi_{s}\right)$$

where, k=2π/λ, λ is the wavelength, $\left(x_{n},y_{n}\right)$ is the location of element *n*, $\theta_{s}$ and $\phi_{s}$ are the elevation and azimuth angle of the desired scanned beam direction, respectively.

Here we investigate 1-D and 2-D scanning performance of the arrays by studying scanning in the elevation plane at *ϕ* = 0° and *ϕ* = 45° directions, but similar results are observed for other scan directions. We note that scanning is essentially shifting the observation window. With sparse arrays this means that if grating lobes are created, scanning may result in more grating lobes appearing in the visible space.

**Figure 9:**
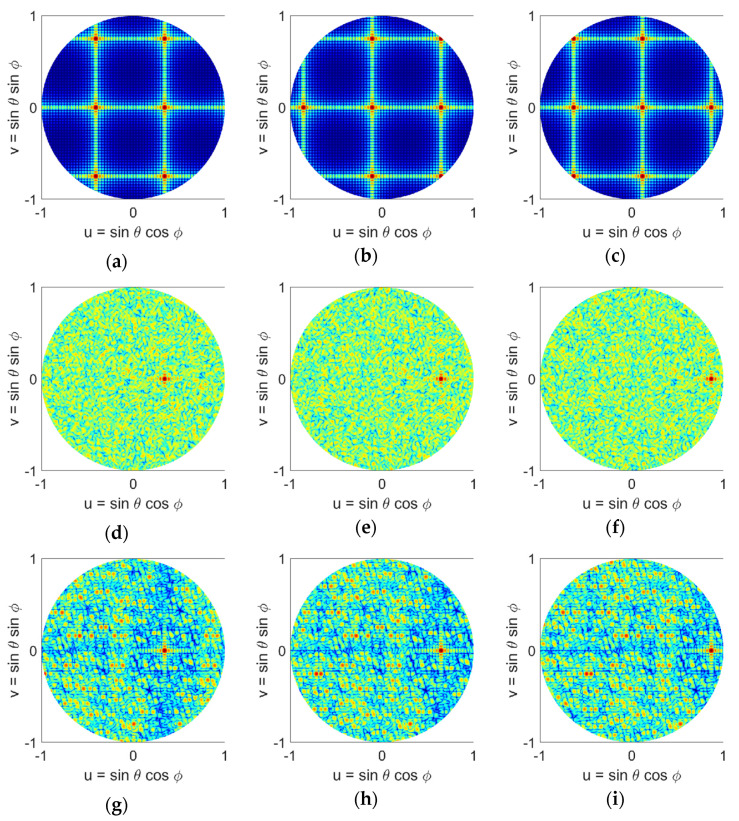

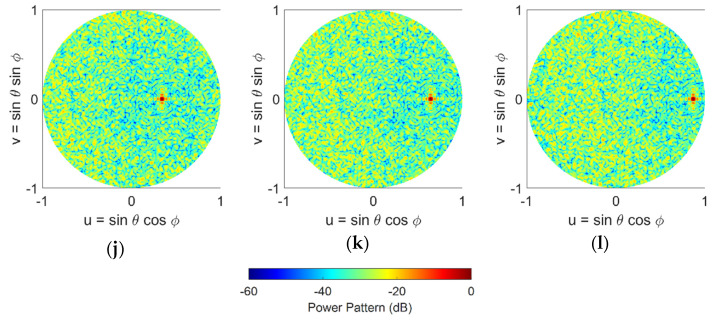
Normalized power patterns of the antenna arrays in the u-v space scanned along the elevation plane in *ϕ* = 0° direction with different element distributions: (**a**) uniform (20° scan), (**b**) uniform (40° scan), (**c**) uniform (60° scan), (**d**) random (20° scan), (**e**) random (40° scan), (**f**) random (60° scan), (**g**) Halton (20° scan), (**h**) Halton (40° scan), (**i**) Halton (60° scan), (**j**) Poisson disk (20° scan), (**k**) Poisson disk (40° scan), (**l**) Poisson disk (60° scan). The Halton sampling uses prime bases of 2 and 7.

**Figure 10:**
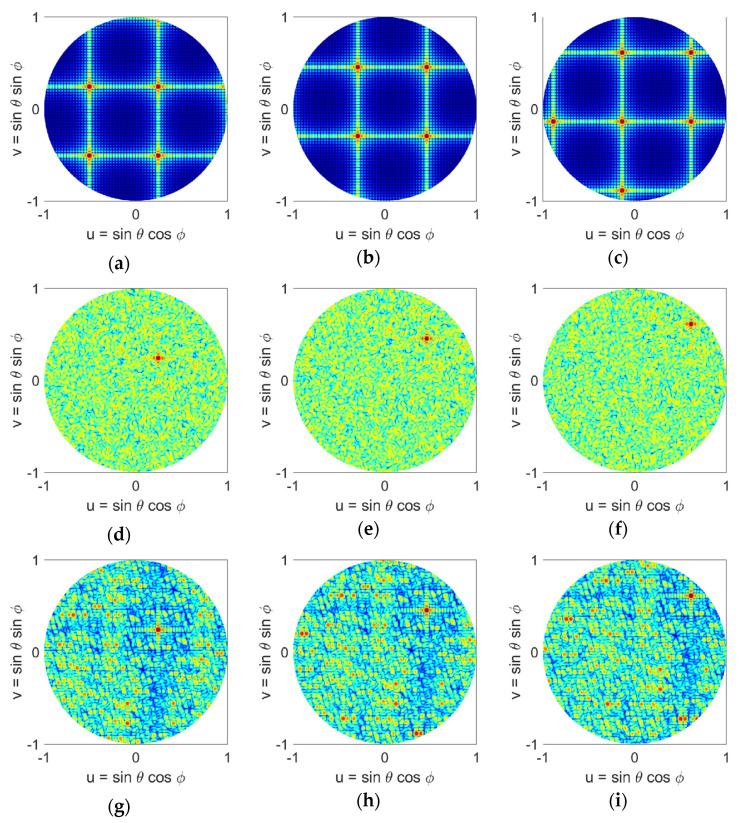

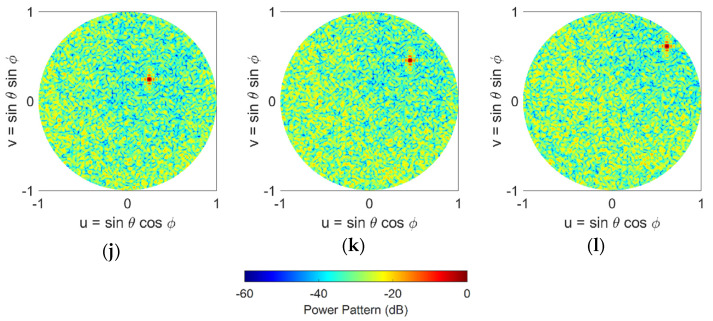
Normalized power patterns of the antenna arrays in the u-v space scanned along the elevation plane in *ϕ* = 45° direction with different element distributions: (**a**) uniform (20° scan), (**b**) uniform (40° scan), (**c**) uniform (60° scan), (**d**) random (20° scan), (**e**) random (40° scan), (**f**) random (60° scan), (**g**) Halton (20° scan), (**h**) Halton (40° scan), (**i**) Halton (60° scan), (**j**) Poisson disk (20° scan), (**k**) Poisson disk (40° scan), (**l**) Poisson disk (60° scan). The Halton sampling uses prime bases of 2 and 7.

Here we compare the scan performance of two LDS sparse arrays that showed the best performance, namely Halton (with bases of 2 and 7), and Poisson distributions, along with uniform and random distributions. Scanned patterns of each of these arrays are given in Figure 9 (*ϕ* = 0° direction) and 10 (*ϕ* = 45° direction), for 20°, 40°, and 60° elevation scans. We note that the array elements are isotropic point sources. For the uniform array, four grating lobes are observed when the array beam is at boresight, Figure 5a. When the array is scanned in 1-D, Figure 9a–c, the number of grating lobes increases to five at 20° and to seven when pointing at 40° and 60°. When the array is scanned in 2-D, Figure 10a–c, the number of grating lobes first reduces to three at 20° and 40°, and then increases to five when pointing at 60°. This change in the number of grating lobes degrades directivity, and in general the uniform array is not suitable for beam-scanning. The other three arrays however, i.e., random, Halton, and Poisson, show a good beam-scanning performance. These arrays do not have grating lobes, and scanning does not change that. In particular, we note that Poisson distribution, Figure 9 and Figure 10j–l, shows a performance better than random distribution, i.e., Figure 9 and Figure 10d–f, with the added advantage of having a physically realizable element distribution. It is important to note that while Halton sampling does have a higher SLL in the visible space, it does not have grating lobe issues, and again provides a notably better element distribution compared to the random case. Nonetheless, these studies also confirm that the Poisson disk sampling provides the best performance.

## 6. Conclusions

Sparse arrays have a significantly smaller number of elements compared to traditional dense arrays (with λ/2 element spacing) which minimizes their cost and complexity. A comprehensive study of sparse phased arrays using low-discrepancy sequence (LDS) element distribution is presented. We show that these LDS element distributions remove the grating lobes associated with large element spacing in sparse arrays, while at the same time avoid undesirable and impractical element distributions in random arrays. The mathematical formulation for implementing LDS for sparse planar arrays is presented, along with numerical studies on their performance. Our studies considered sparse arrays with 86% less elements than a fully populated array. The performance factors compared equidistribution on the aperture of the array by looking at average minimum element spacing, as well as array pattern peak SLL, directivity, and aperture efficiency. Different array aperture configurations were also studied, and we show that the performance of LDS distributions is not impacted by the type/shape of the array. Our studies concluded that in comparison between the LDS techniques, the Poisson disk sampling technique outperforms all other approaches and is the recommended LDS technique for sparse arrays.

## Figures and Tables

**Table 1:** Performance metrics for sparse arrays with different element distributions.

Method	Average Minimum Element Spacing (λ)	Peak SLL (dB)	Directivity (dB)	Aperture Efficiency (%)
Uniform	1.3333	0	31.5478	11.168
Random	0.6667	−10.90	31.9619	12.209
Random with Jitter	0.9325	−9.36	32.5775	14.068
Hammersley (base 2)	1.0037	−7.0	32.7075	14.166
Hammersley (base 3)	1.1688	−2.69	32.6226	14.215
Hammersley (base 5)	1.2624	−0.55	31.6892	11.466
Hammersley (base 7)	0.7538	−0.25	33.8949	19.054
Halton (bases 2, 3)	0.8436	−10.10	32.4609	13.696
Halton (bases 2, 5)	0.8115	−4.33	32.3534	13.361
Halton (bases 2, 7)	0.9172	−12.35	32.8188	14.872
Halton (bases 3, 5)	0.8430	−8.10	32.8611	15.018
Halton (bases 3, 7)	0.7663	−5.90	32.2561	13.065
Halton (bases 5, 7)	0.8633	−5.60	32.9511	15.332
Sobol	0.9307	−6.58	32.7127	14.513
Poisson Disk	0.9031	−12.28	32.8212	14.880

**Table 2:** Performance metrics for sparse antenna arrays with rectangular, circular, and elliptical apertures and different element distributions.

Method/Aperture Type	Average Minimum Element Spacing (*λ*)	Peak SLL (dB)	Directivity (dB)	Aperture Efficiency (%)
Uniform/Rectangular	1.3333	0	31.5778	11.175
Uniform/Circular	1.3333	0	31.5848	11.261
Uniform/Elliptical	1.3333	0	31.5181	11.228
Halton/Rectangular	0.8481	−6.74	32.4857	13.774
Halton/Circular	0.8967	−9.27	32.6474	14.383
Halton/Elliptical	0.7724	−8.94	32.5883	14.365
Poisson Disc/Rectangular	0.9094	−12.18	32.8062	14.829
Poisson Disc/Circular	0.9016	−15.30	32.8643	15.119
Poisson Disc/Elliptical	0.9245	−14.34	32.6634	14.616
